# Editorial: Antidepressant mechanisms of natural products based on multi-omics technologies

**DOI:** 10.3389/fphar.2023.1135791

**Published:** 2023-02-03

**Authors:** Yichao Fang, Tingting Zhou

**Affiliations:** ^1^ School of Pharmacy, Second Military Medical University, Shanghai, China; ^2^ Shanghai Key Laboratory for Pharmaceutical Metabolite Research, School of Pharmacy, Second Military Medical University, Shanghai, China

**Keywords:** antidepressant, multi-omics technologies, natural products, metabolomics, proteomics, genomics, transcriptomics

Depression has become currently one of the leading causes of disease burden in the world leading to suicide and ischemic heart disease. Current antidepressant drugs are not universally effective and have significant side effects. For more than 2,500 years, traditional Chinese medicines (TCM) have played an important role in the treatment of diseases associated with depression. With the modernization of traditional Chinese medicine, some TCMs and metabolites have been validated to alleviate these symptoms and are therefore considered effective in treating depression. This Research Topic aims to investigate the antidepressant mechanisms of metabolites using multi omics approaches including genomics, transcriptomics, proteomics and metabolomics.

Menopausal depression plagues the later life of many women. *Cyperus rotundus* L. [Cyperaceae; Cyperi rhizoma] and *Perilla frutescens* (L.) Britt. [Lamiaceae; Perillae folium] comprise a traditional Chinese botanical drug pair (XS). Li et al. first performed gas chromatography–mass spectrometry (GC-MS) detection of volatile oil of XS, subjected the identified active components to network pharmacology screening, and screened a total of 38 active compounds and 42 overlapping genes, and found that solute carrier family six-member 4 (SLC6A4) and solute carrier family six-member 3 (SLC6A3) regulate serotonergic and dopaminergic synapses and are associated with the antidepressant mechanism of volatile oil of XS. After that, a rat model of menopausal depression was established by chronic unpredictable mild stress (CUMS) and ovariectomy, which verified that XS volatile oil could reverse the depressive behavioral parameters of ovariectomy rats. At the same time, the metabolomics results indicated that XS volatile oil mainly acted on the metabolic pathways regulating phenylalanine, tyrosine and tryptophan biosynthesis, tyrosine metabolism and tryptophan metabolism, which were consistent with the prediction results of network pharmacology.

*Gardenia jasminoides* Ellis [Rubiaceae; Gardeniae fructus] and *Glycine max* (L.) Merr. [Fabaceae; Sojae semen praeparatum] can be composed of a medicinal pair (ZZCD) recorded in Treatise on Febrile Diseases. Zhang et al. found that ZZCD was neuroprotective and could reverse depressive behavior in CUMS rats in their previous study. In this study, Zhang et al. analyzed the cerebral metabolic profiles of CUMS rats by ultra-performance liquid chromatography-quadrupole/time-of-flight mass (UHPLC-Q-TOF/MS) method, and a total of 26 differential metabolites and six differential metabolic pathways were screened. After that they focused on the glutathione metabolic pathway and analyzed the key metabolites in the pathway. Reactive oxygen species, superoxide dismutase, glutathione reductase, and glutathione peroxidase were also measured in the brains of depressed rats. The present study indicated that the antidepressant effects of ZZCD may be associated with regulating the glutathione imbalance and oxidative stress of the brain.

*Bupleurum chinense* DC. [Apiaceae; Bupleuri radix] and *Paeonia lactiflora* Pall. [Ranunculaceae; Paeoniae radix alba] make up the most accepted botanical drug pairs (CBHP) in many classic antidepressant prescriptions. Chen et al. established a CUMS rat model to evaluate the efficacy of CBHP, then performed a metabolomics analysis of the cortex of rats using ultra-high-performance liquid chromatography combined with quadrupole orbitrap mass spectrometry (UPLC-Q-Orbitrap/MS), a total of 21 differential metabolites associated with depression were screened, and the key metabolites in the purine metabolism pathway were quantitatively validated, and found that the CUMS group had disorders of purine metabolism, and the effects of combined administration were better than those of single administration. In addition, this study revealed that CBHP modulated purine metabolism in association with the suppression of malondialdehyde (MDA) production, nod like receptor protein 3 (NLRP3) inflammasome expression, and interleukin (IL)-1β, IL-6, and tumor necrosis factor (TNF)-α related secretion.

Lei et al. focused on paeoniflorin, a metabolite, to establish a reliable depression model using chronic unpredictable mild irritation combined with solitary rearing method, investigated the antidepressant mechanism of paeoniflorin using a combination of rat urine metabolomics and network pharmacology, identified 56 biomarkers, and selected citric acid, thiamine monophosphate, gluconolactone, 5-hydroxyindoleacetic acid, and stachyose were used as key markers to identify SLC6A4, TNF, IL-6 and SLC6A3 as key targets. The binding activity of key targets with paeoniflorin was later verified by molecular docking. This study demonstrates for the first time that paeoniflorin regulates multiple biological pathways and metabolic profiles to alleviate depressive symptoms by affecting key target proteins.

These four studies provide valuable ideas and experiences for exploring the antidepressant mechanisms of natural products, and make a great contribution to the further development of natural product antidepressant drugs. However, several questions remain. For example, these four studies all screened for differential metabolites associated with depression by metabolomics, but the VIP values at screening were not the same, which may have implications for the metabolic pathways finally screened, thus limiting future research directions.

Botanical drug is a complex system, and the mechanism of depression is also very complex. It is therefore highly likely that errors, such as *a priori* validation of effects, will occur during the course of the study. A fundamental characteristic of any scientific method is to base one’s work on a research question or a hypothesis. In order to explore the antidepressant mechanisms of natural products more comprehensively and precisely, the research methods of multi omics combination are increasingly important. The multi omics combination can not only elucidate the antidepressant mechanism of natural products more comprehensively, but also exclude the errors caused by some different experimental conditions, which is more conducive to scientific and precise research.

